# High resolution magnetic resonance imaging in pathogenesis diagnosis of single lenticulostriate infarction with nonstenotic middle cerebral artery, a retrospective study

**DOI:** 10.1186/s12883-018-1054-z

**Published:** 2018-04-25

**Authors:** Li-Li Sun, Zhong-Hao Li, Wen-Xiong Tang, Lei Liu, Fei-Yan Chang, Xue-Bin Zhang, Wei-Jie Ye, Shuo Lu, Zun-Jing Liu, Xian-Jin Zhu

**Affiliations:** 1 Department of Neurology, China-Japan Friendship Hospital, 2 Yinghua Dongjie, Hepingli, Beijing, 100029 China; 2 Department of Radiology, China-Japan Friendship Hospital, 2 Yinghua Dongjie, Hepingli, Beijing, 100029 China

**Keywords:** Lenticulostriate infarction, Branch atheromatous disease, Small vessel disease, High-resolution magnetic resonance imaging, Pathogenesis

## Abstract

**Background:**

It is usually difficult to identify stroke pathogenesis for single lenticulostriate infarction with nonstenotic middle cerebral artery (MCA). Our aim is to differentiate the two pathogeneses, non-branch atheromatous small vessel disease and branch atheromatous disease (BAD) by high-resolution magnetic resonance imaging (HR-MRI).

**Methods:**

Thirty-two single lenticulostriate infarction patients with nonstenotic MCA admitted to the China-Japan Friendship Hospital from December 2014 to August 2017 were enrolled for retrospective analysis. National Institutes of Health Stroke Scale (NIHSS), modified Rankin Scale (mRS), atherosclerotic risk factors, imaging features, and the characteristic of MCA vessel wall in HR-MRI were evaluated.

**Results:**

MCA plaques were detected in 15(46.9%) patients which implied BAD and 8 of 15 (53.3%) patients had plaques location in upper dorsal side of the vessel wall. Patients with HR-MRI identified plaques had a significantly larger infarction lesion length (1.95 ± 0.86 cm versus 1.38 ± 0.55 cm; *P* = 0.031) and larger lesion volume (2.95 ± 3.94 cm^3^ versus 0.90 ± 0.94 cm^3^; *P* = 0.027) than patients without plaques. Patients with HR-MRI identified plaques had a significant higher percentage of proximal lesions than patients without plaques (*P* = 0.055). However, according to the location of MCA plaques, there were no significant differences in terms of imaging features, NIHSS and mRS.

**Conclusion:**

We demonstrated high frequency of MCA atheromatous plaques visualized in single lenticulostriate infarction patients with nonstenotic MCA by using HR-MRI. Patients with HR-MRI identified plaque presented larger infarction lesions and more proximal lesions than patients without plaque, which were consistent with imaging features of BAD. HR-MRI is an important and effective tool for identifying stroke etiology in patients with nonstenotic MCA.

## Background

Stroke is an important public health problem throughout the world with major mortality and severe morbidity. The accurate identification of stroke etiology is important for guiding patient management and prognosis [1]. Lenticulostriate infarction is ischemia in the territory supplied by the deep perforating branches of the middle cerebral artery (MCA). Lenticulostriate arteries supply the basal ganglia and most of the internal capsule [2]. There are two different vascular pathogenesis in single lenticulostriate infarction with nonstenotic MCA: 1) branch atheromatous disease (BAD), atheromatous plaque of MCA at the orifice of lenticulostriate arteries and 2) non-branch atheromatous small vessel disease, histologically characterized by lipohyalinotic degeneration of lenticulostriate arteries themselves [3]. BAD was proposed by Caplan in 1989 [4]. It was identified as a new pathogenesis caused by an occlusion or stenosis at the origin of a deep penetrating artery of the brain. Non-branch atheromatous small vessel disease is different from BAD showed lower mortality and higher risk of death when with anticoagulant therapy [1].

However, it is usually difficult to distinguish BAD from non-branch atheromatous small vessel disease for practicing clinicians. Conventional imaging techniques, magnetic resonance angiography (MRA), computed tomography angiogram (CTA), and digital subtraction angiography (DSA) can’t identify the two pathogenesis in nonstenotic arteries because those only show the information of the lumen, not the vessel wall.

High-resolution magnetic resonance imaging (HR-MRI) could display the features of arterial wall, and recent studies showed that it was also an effective tool for identifying plaque of intracranial arteries [5–7]. In this study, we aimed to determine whether HR-MRI can identify early stage atherosclerotic plaque in patients with nonstenotic MCA on MRA, and investigate the clinical distinction of the two pathogenesis BAD and non-branch atheromatous small vessel disease.

## Methods

### Patients

Between December 2014 and August 2017, patients from China-Japan Friendship Hospital met the following criteria were enrolled: (1) a single new-onset lenticulostriate artery territory infarction on diffusion weighted imaging (DWI); and (2) no relevant MCA disease on MRA. Patients with the following conditions were excluded: (1) had a definite cardioembolic source (e.g., atrial fibrillation, recent myocardial infarction, dilated cardiomyopathy, valvular heart disease, and infectious endocarditis); (2) stenosis of ipsilateral internal carotid artery; or (3) nonatherosclerotic vasculopathy (e.g., dissection, arteritis, and moyamoya disease).

For all enrolled patients, age, gender, atherosclerotic risk factors including hypertension (defined as receiving medication for hypertension or blood pressure > 140/90 mmHg on repeated measurements), diabetes mellitus (defined as receiving medication for diabetes mellitus, fasting blood glucose ≧126 mg/dL, and/or 2-h postprandial blood glucose ≧200 mg/dL), hyperlipidemia (defined as receiving cholesterol reducing agents or overnight fasting cholesterol level > 200 mg/dL), hyperhomocysteinemia (defined as serum level of total homocysteine ≥15 μmol/L), tobacco consumption, and history of stroke and coronary heart disease were collected. National Institutes of Health Stroke Scale (NIHSS) score was measured at the time of admission and discharge. Modified Rankin Scale (mRS) score was measured at the time of discharge. All patients underwent 24 h of electrocardiographic monitoring and transthoracic echocardiography to exclude silent cardioembolism.

### Imaging protocol

Brain MRI examinations were performed in a 3 T scanner (Ingenia; Philips Healthcare, Best, the Nederland) with a 15-channel phased-array head coil. Axial T2-weighted, fluid-attenuated inversion recovery and DWI were obtained to evaluate infarction lesions. All above sequences had 5 mm slice thickness and 1.0 mm interstice gap. 3D time-of-flight (TOF) MRA was obtained with the following parameters: repetition time/echo time = 21 ms/3.2 ms, field of view (FOV) =200 × 200 × 344 mm3, matrix = 400 × 287 × 287, and number of signal averages (NSA) =1. Acquisition (ACQ) voxel volume was 0.5 × 0.7 × 1.2 mm3. Reconstruction (REC) voxel volume was 0.5 × 0.5 × 0.6 mm3. 3D volumetric isotropic turbo spin echo acquisition (VISTA) images were obtained with the following parameters: repetition time/echo time = 1300 ms/36 ms, FOV = 140 × 200 × 135 mm3, matrix = 280 × 332 × 270, NSA = 2. ACQ voxel volume was 0.5 × 0.6 × 0.5 mm3. REC voxel volume was 0.5 × 0.5 × 0.5 mm3. The short axial cross-sections were constructed automatically with 0.5 mm slice thickness. MR images were transferred to a digital picture archiving and communication (PACS) workstation. MCA plaques were assessed on both short axial and long axial images. The location of plaques and the relationship between plaques and infarcts were evaluated carefully.

Based on fluid-attenuated inversion recovery imaging, leukoaraiosis was graded from 1 to 3 according to the visual Fazekas scale:grade 1, mild (single lesions< 10 mm; areas of “grouped” lesions< 20 mm in any diameter); grade 2, moderate (single hyperintense lesions between 10 to 20 mm; areas of “grouped” lesions≥20 mm in any diameter; no more than “connecting bridges” between individual lesions); and grade 3, severe (single lesions or confluent areas of hyperintensity ≥20 mm in any diameter) [8]. Fazekas scale of deep white matter changes with scores of 2 and 3 considered significant leukoaraiosis.

**Fig. 1:**
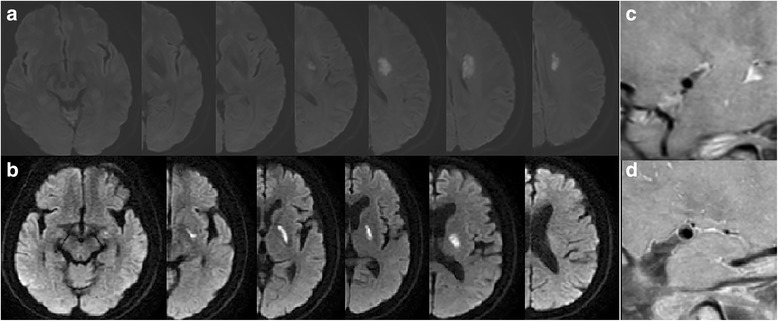
Two types of single lenticulostriate artery territory infarction with no relevant MCA disease on MRA: (**a**) Distal single lenticulostriate artery territory infarction lesion (not extending to the basal surface of the MCA), (**b**) Proximal single lenticulostriate artery territory infarction lesion (extending to the basal surface of MCA), (**c**) HRMRI showed no plaque on MCA, (**d**) HRMRI showed plaque on MCA

Based on DWI, the largest lesion was used to determine the volume of infarction: 1/2 × diameter of length × diameter of width × (0.5 × numbers of DWI slices of acute infarction). Lesion location in relation to the parent artery (MCA) was dichotomized as proximal infarction lesion (extending to the basal surface of the parent artery) and distal infarction lesion (not extending to the basal surface of the parent artery; Fig. 1) [9].

### Statistical analysis

Continuous values were expressed as mean ± SD and nominal variables as count and percentages. Median values and the interquartile range (IQR) were computed for nonnormally distributed variables. The Fishers exact test or χ^2^ test was performed to assess the categorical variables. The normally distributed continuous variables were analyzed by independent sample *t*-test. Continuous variables nonnormally distributed were analyzed by Mann-Whitney U-test. Statistical analyses were conducted using SPSS for Windows (version 20.0). A 2-sided value of *P* < 0.05 was considered to indicate a statistically significant difference.

## Results

### General patient characteristics

During the study period, a total of 32 patients (10 female and 22 male) were enrolled. The mean age was 60.97 ± 11.54. Atherosclerotic risk factors included hypertension in 22 patients (69.0%), diabetes mellitus in 8 patients (25%), hyperlipidemia in 20 patients (62.5%), hyperhomocysteinemia in 7 patients (21.9%), and tobacco consumption in 10 patients (31.3%). 3 patients (9.4%) had histories of stroke and 0 patient had histories of coronary heart disease. The median NIHSS score at admission was 2.5 (IQR: 1.0 to 4.0). The median NIHSS score at discharge was 1.5 (IQR: 1.0 to 3.75). The median mRS score at discharge was 1.0 (IQR: 0 to 2.0).

MCA plaques were detected in 15 (46.9%) patients and, of these, 8 (53.3%) patients had plaques’ location involved upper dorsally part of the vessel wall (Table 1). The mean infarction lesion length was 1.64 ± 0.76 cm. The median number of infarction lesion slices was 3.0(IQR: 2.0 to 4.0). The mean infarction lesion volume was 1.86 ± 2.92 cm^3^. The number of patients with proximal infarction lesion was 20(62.5%). 19(59.4%) patients had significant leukoaraiosis (Fazekas grade≧2).

**Table 1:** VISTA images of all enrolled patients

Non-Plaque	Plaque		Total
17 (53.1%)	15 (46.9%)		32
	Location involving dorsally upper part	Location not involving dorsally upper part	
	8 (53.3%)	7 (46.7%)	

*VISTA* volumetric isotropic turbo spin echo acquisition

### Comparison of patient characteristics between patients with and without HR-MRI identified plaques

The plaque group had a higher percentage of diabetes mellitus than non-plaque group (7[46.7%] versus 1[5.9%]; *P* = 0.013). No other significant differences were found in terms of demographic characteristics, atherosclerotic risk factors, NIHSS and mRS between the two groups (Table 2). On the contrast to the non-plaque group, the plaque group had a significantly larger infarction lesion length (1.95 ± 0.86 versus 1.38 ± 0.55 cm; *P* = 0.031). Besides, the lesion volume was significantly larger in patients with plaque than in patients without plaque (2.95 ± 3.94 versus 0.90 ± 0.94 cm^3^; *P* = 0.027). Of the 32 patients, the median number of infarction lesion slices was 3.0. Patients were divided into two groups: patients with number of lesion slices ≧ 3.0 and patients with number of lesion slices < 3.0. The plaque group had more patients with number of lesion slices ≧ 3.0 than non-plaque group with a borderline significance (*P* = 0.055). On the contrast to non-plaque group, the plaque group had a higher percentage of proximal lesions with a borderline significance (*P* = 0.055). There were no significant differences in terms of significant leukoaraiosis (Fazekas grade≧2).

**Table 2:** Patient Characteristics According to the Presence of MCA Plaques

Characteristics	Plaque (*n* = 15)	Non-Plaque (*n* = 17)	*P* value
Demographics			
Age, years, mean ± SD	63.47 ± 10.00	58.76 ± 12.63	0.257
Male, n(%)	10(66.7%)	12(70.6%)	0.811
Atherosclerotic risk factors			
Hypertension, n (%)	12(80.0%)	10(58.8%)	0.197
Hyperlipidemia, n (%)	9(60.0%)	11(64.7%)	0.784
Diabetes mellitus, n (%)	7(46.7%)	1(5.9%)	0.013
Hyperhomocysteinemias, n (%)	2(13.3%)	5(29.4%)	0.402
Previous history of stroke, n (%)	2(13.3%)	1(5.9%)	0.589
Tobacco consumption, n (%)	3(20.0%)	7(41.2%)	0.197
Imaging features			
Lesion length, cm (mean ± SD)	1.95 ± 0.86	1.38 ± 0.55	0.031
Number of lesion slices (median[IQR])	3.0(3.0–5.0)	2.0(2.0–3.5)	0.132
Number of lesion slices ≧ 3, n (%)	12(80.0%)	8(47.1%)	0.055
Lesion volume, cm^3^ (mean ± SD)	2.95 ± 3.94	0.90 ± 0.94	0.027
Proximal lesion, n (%)	12(80.0%)	8(47.1%)	0.055
Significant leukoaraiosis (Fazekas grade ≧ 2), n (%)	11(73.3%)	8(47.1%)	0.131
Severity of stroke			
NIHSS at admission(median[IQR])	2.0(1.0–4.0)	3.0(1.0–5.5)	0.278
NIHSS at discharge(median[IQR])	1.0(0–3.0)	2.0(1.0–4.0)	0.628
mRS ≧ 2, n (%)	4(26.7%)	5(29.4%)	1.000

*MCA* middle cerebral artery, *SD* standard deviation, *IQR* interquartile range, *NIHSS* National Institutes of Health Stroke Scale, *mRS* modified Rankin Scale

### Comparison of patient characteristics according to the location of MCA plaques

**Fig. 2:**
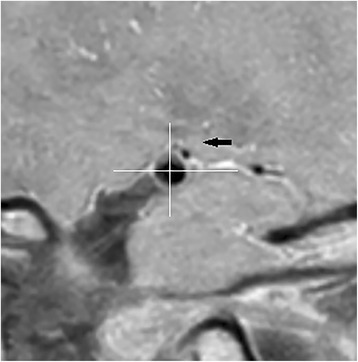
Example of plaque location involving dorsally upper part. The black arrow showed the dorsally upper part

We divided patients with HR-MRI identified MCA plaque into two groups: plaque location involving upper dorsally part and plaque location not involving upper dorsally part (Fig. 2). There were no significant differences in terms of imaging and clinical features (Table 3).

**Table 3:** Patient Characteristics According to the Location of MCA Plaques

	Plaque location involving dorsally upper part (*n* = 8)	Plaque location not involving dorsally upper part (*n* = 7)	*P* Value
Imaging features			
Lesion length, cm (mean ± SD)	1.86 ± 0.81	2.05 ± 0.96	0.686
Number of lesion slices (median[IQR])	3.0(3.0–4.75)	3.0(2.0–5.0)	0.779
Number of lesion slices ≧ 3, n (%)	7(87.5%)	5(71.4%)	0.569
Lesion volume, cm^3^ (mean ± SD)	3.47 ± 5.10	2.37 ± 2.25	0.955
Proximal lesion	7(87.5%)	5(71.4%)	0.569
Significant leukoaraiosis (Fazekas grade ≧ 2), n (%)	6(75.0%)	5(71.4%)	1.000
Severity of stroke			
NIHSS at admission	2.0(1.0–3.75)	1.0(0–7.0)	0.867
NIHSS at discharge	1.5(1.0–2.75)	1.0(0–5.0)	1.000
mRS ≧ 2	2(25%)	2(28.6%)	1.000

*MCA* middle cerebral artery, *SD* standard deviation, *IQR* interquartile range, *NIHSS* National Institutes of Health Stroke Scale, *mRS* modified Rankin Scale

## Discussion

It is usually difficult to distinguish BAD and non-branch atheromatous small vessel disease for practicing clinicians. Microatheromas have been reported to be the most common underlying mechanism of symptomatic lacunar infarction in autopsy studies [10]. However, traditional angiography examinations, such as MRA, CTA and DSA, cannot identify BAD. Recent studies have reported HR-MRI is an effective and directive tool for the evaluation of intracranial atherosclerotic plaques. In our study, we found that as many as 46.9% of acute lenticulostriate stroke patients with nonstenotic MCA on MRA demonstrated HR-MRI identified MCA plaques. This result is consistent with previous studies about MCA plaques in patients with lacunar infarction [11–13].

**Fig. 3:**
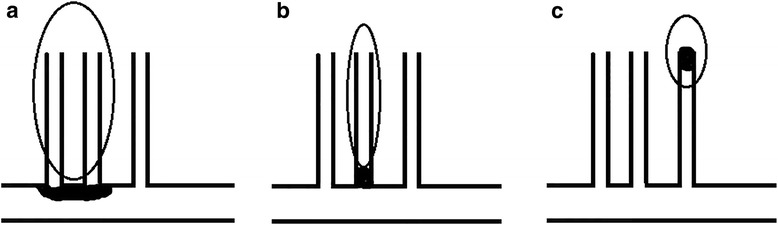
Mechanisms of single lenticulostriate infarction: (**a**) Large proximal infarction lesion caused by plaque within parent artery(MCA) blocking the lenticulostriate artery orifice; (**b**) Proximal infarction lesion caused by microatheroma in the orifice of the lenticulostriate artery itself; (**c**) Distal lacunar infarction lesion caused by small vessel occlusion

Previously identification of BAD is mainly based on the neuroimaging features on traditional brain MRI. However, different studies used different methods to define BAD. Yamamoto et al. defined BAD of the lenticulostriate stroke as infarcts more than 10 mm in diameter on axial slice and visible on 3 or more axial slices [3]. Jeong et al. defined BAD as lesions visible on 4 or more axial slices [14]. Nah et al. considered that infarction lesion involvement of the lowest portion of the basal ganglia was an indication of BAD [9]. A study from China, 312 patients with lacunar infarction who had normal MCA on MRA enrolled, suggested that infarct lesion size may be used as a method to identify BAD [15]. According to the studies mentioned above, BAD tended to have larger infarcts and lesions were closer to the orifice of the perforating artery. However, these studies lacked directive atherosclerotic plaques evidences on HR-MRI. In our study, we showed that patients with HR-MRI identified plaque had larger infarction lesions (*P* < 0.05) and tended to present proximal lesions (*P* = 0.055) and ≧3 lesion slices (*P* = 0.055), which was consistent with the past studies about imaging features of BAD. Lenticulostriate artery originate from the initial segment of MCA, BAD tend to lead large proximal infarction lesion we think it was caused by plaque within parent artery (MCA) blocking the lenticulostriate artery orifice (Fig. 3a). But we can’t directly show the plaque and the orifice of perforator branches in HR-MRI due to the perivascular space or Virchow-Robin spaces. Kim et al. found that located plaque, and plaque length no related to large lenticulostriate infarction. And considered that it was due to the variation of branching pattern of perforators, which occlusion of a single lenticulostriate artery produced a variety of infarct sizes [16, 17]. In fact, both of the two pathogenesis can lead to the large infarction, we need a better way to show the details of lenticulostriate artery from orifice to ending, which can be used to distinguish the two pathogenesis.

However, the frequencies of proximal infarction lesions were not significantly different between patients with plaque and without plaque. We speculated that this result was associated with some patients had microatheroma in the orifice of the lenticulostriate artery itself (Fig. 3). In this hypothesis, patients could have proximal infarction lesions, but without HR-MRI identified MCA plaque. Different from a group lenticulostriate arteries occluded by MCA atherosclerotic plaque, this group patients could present small infarction lesions. So we furtherly speculated that microatheroma in the orifice of the lenticulostriate artery itself was the possible pathogenesis of small proximal infarction lesion. Unfortunately, microatheroma in the orifice of the lenticulostriate artery itself could not identified directly by using HR-MRI. Besides, the branching patterns of MCA perforators vary greatly in terms of type [18, 19] and infarction size and location may also be related to these variations.

What’s more, in our study 10 patients’ infarction lengths were more than 20 mm which can defined as large lenticulostriate infarction, and of these 6 patients with HR-MRI identified plaque. In the Trial of ORG 10172 in Acute Stroke Treatment (TOAST) criteria, lenticulostriate infarction which length less than 15 mm was belong to the smell-vessel occlusion subtype [20]. But several studies found nearly half of patients with small vessel disease suffered infarction more than 15 mm [16], and small vessel disease can lead to an infarction with length even more than 20 mm because of occlusion of multiple perforating arterioles [21]. New ischemic stroke classifications included lesions of up to 20 mm in diameter [22] or even abandoned the rule of length of infarction [23, 24].

Theoretically, non-branch atheromatous small vessel disease is more strongly associated with hypertension and leukoaraiosis. Nevertheless, Yamamoto et al. found that there were no significant differences in prevalence of hypertension, diabetes mellitus, and hyperlipidemia between non-branch atheromatous small vessel disease and BAD [3]. In our study, we showed higher prevalence of diabetes mellitus in patients with MCA plaques than patients without MCA plaques. However, there were no differences in terms of other risk factors of atherosclerosis and significant leukoaraiosis between two groups.

Microanatomy studies suggested that most of penetrating arteries arose dorsally from the upper part of MCA wall [19]. We subdivided patients with MCA plaque into two groups: plaque location involving dorsally upper part and plaque location not involving dorsally upper part. However, there were no significant differences in terms of imaging features and severity of stroke between the two groups. We attributed this partly to the limited number of enrolled patients in our study and partly to the variations of branching patterns of MCA perforators [19].

## Conclusion

Pathogenesis diagnosis of ischemic stroke is important for patient management. However, it is usually difficult to distinguish BAD from non-branch atheromatous small vessel disease for lenticulostriate infarction patients with nonstenotic MCA by using traditional imaging examinations. In our study, we demonstrated high frequency of MCA atheromatous plaques visualized on HR-MRI in patients with nonstenotic MCA on MRA. Patients with HR-MRI identified plaque presented larger infarction lesions and more proximal lesions than patients without plaque, which were consistent with imaging features of BAD. HR-MRI was an important and effective tool for identifying stroke etiology, especially for patients with normal MCA by MRA. We strongly suggest that HR-MRI is particularly important for large proximal lenticulostriate infarction patients with nonstenotic MCA.

## Abbreviations

ACQ: Acquisition
BAD: branch atheromatous disease
CTA: computed tomography angiogram
DSA: digital subtraction angiography
DWI: diffusion weighted imaging
FOV: field of view
HR-MRI: high resolution magnetic resonance imaging
IQR: interquartile range
LAA: large artery atherosclerosis
MCA: middle cerebral artery
MRA: magnetic resonance angiography
mRS: Modified Rankin Scale
NIHSS: National Institutes of Health Stroke Scale
NSA: number of signal averages
REC: Reconstruction
SD: standard deviation
VISTA: volumetric isotropic turbo spin echo acquisition
